# Epitope selection for combination therapy using antibody-drug conjugates and immune immune-stimulating antibody conjugates

**DOI:** 10.3389/fimmu.2026.1960790

**Published:** 2026-10-08

**Authors:** Hyeyoung Kwon, Anna Kopp, Tiexin Wang, Andrew Philippart, Dong Jun Koo, Haolong Huang, Troyee Naha, Peter M. Tessier, Greg M. Thurber

**Affiliations:** 1 Department of Chemical Engineering, University of Michigan, Ann Arbor, MI, United States; 2 Biointerfaces Institute, University of Michigan, Ann Arbor, MI, United States; 3 Department of Biomedical Engineering, University of Michigan, Ann Arbor, MI, United States; 4 Department of Pharmaceutical Sciences, University of Michigan, Ann Arbor, MI, United States; 5 Rogel Cancer Center, University of Michigan, Ann Arbor, MI, United States

**Keywords:** ADC, drug delivery, ISAC, targeted therapy, TLR7 agonist

## Abstract

**Introduction:**

With an increase in FDA-approved antibody-drug conjugates (ADCs), recent work has focused on potential combination therapies to maximize efficacy, including those with immune-stimulating antibody conjugates (ISACs). When combining antibody therapies against the same target, competitive antibodies can improve tumor tissue distribution and efficacy, while mixtures of non-competitive antibodies can crosslink receptors and increase cellular internalization but reduce tissue penetration.

**Methods:**

Given these trade-offs, we investigated the impact of combination therapy using competitive and non-competitive antibodies on two cytotoxic ADCs – trastuzumab emtansine (T-DM1) and trastuzumab deruxtecan (TDXd) – in combination with trastuzumab (competitive) or pertuzumab (noncompetitive) antibodies with or without a moderate-potency Toll-Like Receptor 7 (TLR7) agonist payload to enable high ISAC dosing.

**Results:**

Competitive trastuzumab antibodies achieved more homogeneous tumor distribution, while non-competitive pertuzumab reduced tissue penetration. However, HER2 receptor downregulation with pertuzumab enhanced HER2 signaling blockade and was associated with increased payload delivery to cancer cells. The combination of a trastuzumab-ISAC to increase tissue penetration and pertuzumab to enhance cellular internalization of the T-DXd resulted in maximum efficacy. Both combinations of T-DXd with a TLR7 ISAC resulted in maximum complete responses.

**Discussion:**

These findings highlight the importance of epitope selection in combination therapy and demonstrate that strategies to increase both tumor tissue penetration and ADC internalization while delivering multiple payloads result in the greatest efficacy of ADC and ISAC combination treatments. The rational design of antibody combinations with competitive and non-competitive epitopes can achieve these aims to maximize tumor growth inhibition and complete responses at tolerable doses.

## Introduction

Antibody-drug conjugates (ADCs) are a rapidly expanding class of targeted therapeutics with over 100 agents at various stages of clinical development (1, 2). ADCs consist of a monoclonal antibody and cytotoxic payload conjugated through a chemical linker that enables targeted delivery of the chemotherapeutic agent to the cancer site. However, ADC monotherapy is often not sufficient, as patients eventually develop resistance to the drug. Key factors contributing to the resistance include reduced sensitivity to the payload, receptor downregulation, heterogeneous antigen expression, defective internalization, upregulation of anti-apoptotic proteins, and increased drug efflux pumps (3–5). In particular, antibodies and ADCs with high affinity to their targets are limited by their perivascular distribution in solid tumors, which is due to the rapid binding of targets relative to tissue penetration (6). This heterogeneous distribution allows untargeted cells to escape therapy, leading to tumor relapses and poor prognosis. While increasing the antibody dose can improve the penetration (7, 8), the maximum tolerated dose (MTD) for highly potent ADCs is constrained by the toxicity of the payload (9–12). Consequently, the search for combination therapies to increase the rate and duration of responses is becoming more prominent.

One strategy to increase the durability of responses is to combine ADCs with immuno-oncology (IO) drugs, as this combination has shown promising results in numerous preclinical and several clinical studies (13–17). The rationale for combining ADC and IO therapies lies in the ability of ADCs to stimulate immune responses via multiple mechanisms (18, 19). Several payloads are able to induce immunogenic cell death (ICD), where dying tumor cells release Damage-Associated Molecular Patterns (DAMPs) to stimulate immune cells, while the antibody backbone can trigger antibody-dependent cellular cytotoxicity (ADCC) and antibody-dependent cellular phagocytosis (ADCP) through Fc interactions with immune cells (14, 20–22). It has also been reported that microtubule-depolymerizing payloads can stimulate dendritic cells (23, 24), which play a significant role in adaptive immune responses. Therefore, immunotherapy is often paired with ADCs in pursuit of greater efficacy at tolerable doses. However, ADC/IO combinations often show the best efficacy in populations sensitive to checkpoint inhibition. To expand responses to less immunogenic tumors, immune-stimulating antibody conjugates (ISACs) have been developed as a novel antibody-mediated therapeutic to directly target cancer and immune cells.

ISACs employ immune-stimulatory agents such as Toll-like receptor (TLR) agonists and stimulators of interferon genes (STING) agonists, activating innate immunity. While variations exist (25), many utilize Fc receptor-mediated internalization following cancer cell binding to deliver the payload into innate immune cells (26). NJH-395 and BDC-1001 are examples of ISACs that use antibodies targeting human epidermal growth factor receptor-2 (HER2) – an antigen overexpressed in certain breast, gastric/gastroesophageal, bladder, head and neck cancers (27) – and are conjugated with novel TLR7 and TLR7/8 agonists (28), respectively. Similar to ADCs, they can suffer from a narrow therapeutic window, and ultrapotent payloads can drive on-target off-tumor toxicity such as cytokine release syndrome or anti-drug antibody responses (29, 30). Analogous to cytotoxic ADCs, high potency payloads may result in toxicity at subtherapeutic doses (e.g., PBD payloads in solid tumors and NJH-395) while low potency payloads may not have sufficient efficacy even at high doses (e.g., methotrexate/doxorubicin ADCs and BDC-1001). Therefore, we selected a TLR7 agonist with intermediate potency compared to the clinically tested ISACs to pair with an ADC in this work (**Supplementary Figure S1**). One strategy for pairing ISACs with ADCs is to utilize the ADC to drive tumor regression while the ISAC directs the immune response for increased durability and complete responses. This combination alleviates the need for aggressive ISAC immune activation to drive tumor regression, instead focusing on durability of response to potentially provide a larger therapeutic window. However, when combined with anti-HER2 ADCs, such as trastuzumab emtansine (T-DM1) or trastuzumab deruxtecan (T-DXd), it remains unclear how multiple targeted therapies against the same antigen will impact efficacy because targeting the same epitope may lead to competition between therapeutics, while targeting different epitopes could result in faster internalization and reduced penetration.

Previous work evaluated the impact of targeting the same versus different epitopes of surface antigens when combining an antibody with ADC treatment (8, 31–33). In solid tumors treated with sub-saturating ADC doses, co-administration of a competitive antibody that targets the same epitope, such as trastuzumab with T-DM1, improved tissue penetration, thus delivering ADC to more cancer cells and improving efficacy (8). In contrast, non-competitive antibodies that target different epitopes can crosslink the receptor and enhance internalization (34). These combinations show varying effects depending on the level of expression and intrinsic internalization (32, 33). For example, a high dose of a well-tolerated ADC against a slow internalizing receptor, such as carcinoembryonic antigen, benefits from faster internalization driven by non-competitive antibody crosslinking (32). However, rapid internalization of a potent ADC given at a lower dose could lead to decreased penetration in a high-expressing tumor, such as prostate-specific membrane antigen (33), thereby reducing efficacy. Therefore, a quantitative understanding of the trade-offs in epitope selection is needed when combining cytotoxic ADCs with immune-stimulating ISACs, specifically regarding competitive combinations that offer better penetration versus non-competitive ones that increase internalization and reduce signaling.

Here, we tested trastuzumab-based ADCs combined with trastuzumab- or pertuzumab-based ISACs to investigate the efficacy of combinations of competitive and non-competitive antibodies. Trastuzumab and pertuzumab both target HER2, but trastuzumab binds to subdomain IV while pertuzumab binds to subdomain II (35). A combination of non-competitive antibodies is known to inhibit HER2 dimerization, which is important in the cell signaling cascade, and effectively internalize HER2 by crosslinking (36, 37). This simultaneously impacts ADC payload distribution in cells and tissue while also impacting levels of surface-bound antibody. Therefore, we tested trastuzumab-based ADCs with unconjugated or conjugated trastuzumab/pertuzumab ISACs *in vitro* and *in vivo* to understand the quantitative effects of tumor distribution on payload efficacy combined with cell signaling blockade of HER2 and antibody Fc-effector function.

## Materials and methods

### Cell culture

HCC1954, NCI-N87, E0771, and J774A.1 cell lines were purchased from ATCC. HEK-Blue mTLR7 (murine TLR7 reporter cells) and Jurkat-Lucia™ NFAT-CD16 (ADCC reporter cells) were obtained from InvivoGen. E0771-HER2 was obtained by transducing E0771 with human full-length HER2 gene in lentiviral particles (G&P Biosciences, LTV-HER2) and selecting HER2+ clones via FACS (31). HCC1954, NCI-N87, and E0771-HER2 cells were cultured in RPMI-1640, and J774A.1 in DMEM, each supplemented with 10% (v/v) FBS, 50 U/mL penicillin, and 50 μg/mL streptomycin. HEK-Blue mTLR7 cells were cultured in DMEM with the same base supplements in addition to 100 μg/mL Normocin™, 10 µg/mL of Blasticidin, and 100 µg/mL of Zeocin^®^. ADCC reporter cells were cultured in IMDM supplemented with 10% FBS, 50 U/mL penicillin, 50 μg/mL streptomycin, 100 µg/mL Normocin™, 10 µg/mL of Blasticidin, and 100 µg/mL of Zeocin^®^. All cells were passaged 2–3 times per week up to passage 30 for HCC1954 and NCI-N87 or below passage 15 for E0771-HER2, J774A.1, HEK-Blue mTLR7 and ADCC reporter cells. All cells were incubated at 37 °C and 5% CO_2_ during cell growth and *in vitro* assays. Mycoplasma testing was performed biannually using MycoStrip (InvivoGen, rep-mysnc-100).

### Antibody and ADC conjugation

Trastuzumab (Herceptin, Genentech), Pertuzumab (Perjeta, Genentech), T-DM1 (Kadcyla, Genentech), and T-DXd (Enhertu, Daiichi-Sankyo) were obtained from the University of Michigan Pharmacy. The eFc-HALA antibody, a variant of trastuzumab with enhanced Fc effector functions (eFc) and reduced HER2 affinity (High Avidity, Low Affinity; HALA), was generated as previously reported (38). Antibody or ADC at 2 mg/mL supplemented with 10% sodium bicarbonate (v/v) was reacted with fluorescent probes at molar ratios of 1.2 (AF488, AF647, AF680, Invitrogen) for 45 minutes at room temperature and purified using P6 Biogel (1 g gel/10 mL PBS), resulting in degree of labeling (DoL) from 0.7 to 1.2. For TLR7 agonist conjugation, the antibodies were first reduced using 2 molar equivalents of TCEP (MilliporeSigma) for 2 h at 37 °C and then reacted with 4.5 molar equivalents of TLR7 maleimide linker (InvivoGen, TLR-887). ISACs were also purified using P6 BioGel, resulting in approximately a drug-antibody ratio (DAR) of 2. DoL and DAR were confirmed with UV-Vis spectroscopy.

### 3D spheroids

3D *in vitro* spheroids were incubated using a custom 384-well hanging drop plate as previously described (32, 39, 40). HCC1954 cells (3,000) suspended in 25 μL of seeding media comprising complete RPMI culture media, 1.2% (w/v) methylcellulose (Dow-Corning), and Matrigel (Thermo Fisher Scientific, CB40234A) were added to a 384-well hanging drop plate. Spheroids were cultured for 7 days. To quantify the impact of trastuzumab and pertuzumab on ADC penetration, the hanging drops were incubated with T-DM1-AF647 with and without HER2-targeting antibodies (trastuzumab or pertuzumab) at a final concentration of 300 nmol/L for ADC and 900 nmol/L for carrier antibody. For examining the pharmacodynamic activity with T-DXd, 200 nmol/L of T-DXd-AF647 with 1000 nmol/L of 1) eFc-HALA antibody, 2) pertuzumab, or 3) eFc-HALA antibody and pertuzumab were incubated. The spheroids were collected after 24 h for T-DM1 treatment and 48 and 72 h for T-DXd treatment. Once the spheroids were extracted, they were washed with PBS, fixed with 4% formaldehyde, frozen in optimal cutting temperature (OCT), and stored at -80 °C until further processing. Frozen blocks were sectioned for histology (10-μm slices), and sections were processed for staining and imaging as described above. Image analysis was performed in MATLAB and ImageJ to generate Euclidean distance maps.

### Co-culture antibody uptake assay

The cell proliferation solutions were prepared by adding 20 μL of DMSO into each vial of CellTrace Violet and Far-Red kits (Invitrogen, C34557A and C3454A). After the growth media was discarded from the cell culture flask, the cells were washed with PBS and then incubated with 1 μL of staining solution per 1 mL of cells for 15 min. Stained cells (20,000) of E0771-HER2 and J774A.1 were plated in 96-well plates. Either 40 nM of ADC surrogate (trastuzumab-AF488) and 120 nM of carrier antibodies (unconjugated trastuzumab or pertuzumab) or 40 nM of unconjugated trastuzumab and 120 nM ISAC surrogates (AF488 conjugated trastuzumab or pertuzumab) were added to the cells and incubated for 4 h. After the co-cultured cells were collected, 0.4% Trypan Blue solution was used to quench non-internalized fluorophore. The cells were analyzed by an Attune NxT flow cytometer. Data analysis was performed using FlowJo software, and the median fluorescence intensity (MFI) was normalized to the DoL.

### ADCC and mTLR7 reporter assay

To quantify the impact of combining trastuzumab with the eFc-HALA antibody or pertuzumab on ADCC activation, an ADCC reporter cell assay was performed. NCI-N87 and ADCC reporter cells were collected and centrifuged at 150 g for 5 min after cell culture. The cell pellets were resuspended in IMDM media with 10% FBS, 50 U/mL penicillin, and 50 μg/mL streptomycin. NCI-N87 cells (100,000) were seeded into 96-well plates, followed by the addition of testing antibodies at different concentrations. The plates were incubated at 37 °C for 1 h before adding 200,000 ADCC reporter cells to the plates. After adding the reporter cells, the plates were incubated for 6 h until the luminescence assay was performed based on the manufacturer’s instructions (QUANTI-Luc reagent, InvivoGen).

To detect the NF-κB signaling pathway activated by TLR7-ISACs, HEK-Blue mTLR7 reporter cells were collected, resuspended in HEK-Blue detection solution, and seeded at 40,000 cells per well in 96-well plates. Cells were incubated with ISACs or control antibodies at a final concentration of 100 nM for 16 h. Fold induction was quantified by measuring absorbance at 650 nm using a BioTek plate reader.

### *In vivo* histology and efficacy study

All animal studies were approved and conducted in accordance with the University of Michigan Institutional Animal Care and Use Committee. For all tumor xenograft studies, 5×10^6^ NCI-N87 cells in 50% (v/v) Matrigel were inoculated in the rear flank of 6–8 weeks old female nude (Foxn1 nu/nu) mice obtained from Jackson Laboratories. For the tumor distribution study, 6.4 mg/kg of fluorescently labeled T-DXd (T-DXd-AF680) with or without naked trastuzumab or pertuzumab at 1:3 ratio (ADC:antibody) was injected through the tail vein once the tumor volume reached ~250 mm^3^ (n = 3/group). Before sacrifice, 15 mg/kg of Hoechst 33342 was injected intravenously to stain nuclei of cells near active blood vessels (to visualize endothelial cells during immunofluorescence imaging). The tumors were collected 24 hours after ADC administration and flash-frozen in OCT for histology. The tumor blocks were sectioned in 10-μm slices using a Leica Cryostat and imaged using an Olympus Confocal Microscope. Images were analyzed using Fiji.

For efficacy studies, the following treatments were administered in NCI-N87 bearing nude mice once via tail vein: 1) 6.4 mg/kg trastuzumab and 32 mg/kg pertuzumab, 2) 32 mg/kg eFc-HALA-TLR7, 3) 32 mg/kg Pertuzumab-TLR7, 4) 6.4 mg/kg T-DXd, 5) 6.4 mg/kg T-DXd and 32 mg/kg of eFc-HALA-TLR7, 6) 6.4 mg/kg T-DXd and 32 mg/kg pertuzumab, and 7) 6.4 mg/kg T-DXd, 32 mg/kg eFc-HALA-TLR7, and 32 mg/kg pertuzumab. Details on efficacy studies are provided in **Supplementary Information** (**Supplementary Tables S1**, **S2**). The tumor volumes were measured every 3 days using a digital caliper and calculated as width^2^ × length ÷ 2. Mice were monitored until the humane endpoint, and data were plotted using GraphPad Prism. Animals that died or were euthanized due to health-concerns were included in the Kaplan-Meier survival analysis as events. Mice that remained alive at the end of the 150-day observation period were censored at Day 150.

### Statistics

All statistical analyses were performed using GraphPad Prism. For comparison of macrophage uptake and tumor volume at 90 days post-treatment, an unpaired t-test was used. For efficacy studies, tumor-growth curves were summarized by calculating the area under the curve (AUC) for each animal. AUC values were compared using one-way ANOVA followed by Tukey’s multiple-comparisons test. For survival analysis, Kaplan–Meier survival curves were compared between the T-DXd group and each combination-treatment group using the Log-rank (Mantel-Cox) test. Statistical significance was defined as P < 0.05.

## Results

### Epitope selection for ADC/antibody combination therapy impacts tissue penetration

ADCs often have limited intratumoral distribution in solid tumors, which is a major driver of efficacy. The competition between ADCs and their corresponding antibodies can enable the ADCs to penetrate deeper into the tissue by partially blocking the perivascular binding sites. In contrast, non-competitive antibodies have been reported to crosslink receptors to form clusters, leading to faster internalization and potentially lower tissue penetration (32, 37, 41, 42). To demonstrate this, we first examined the distribution of T-DM1 alone or with trastuzumab (a competitive antibody) or pertuzumab (a non-competitive antibody) in 3D *in vitro* spheroids to visualize the impact on distribution. HCC1954 cells with high HER2 expression were seeded in a 384-well hanging drop plate and incubated for 7 days to form spheroids. A sub-saturating dose of fluorescently labeled T-DM1 was incubated alone or with either unconjugated trastuzumab or pertuzumab at a 1:3 (ADC:antibody) ratio known to impact tissue penetration (8). When T-DM1 was incubated by itself, the ADC penetrated two to three cell layers from the edge into the spheroid (**Figure 1A**). The addition of trastuzumab enabled the ADC to more homogeneously distribute and reach the center of the spheroids. In contrast, the addition of pertuzumab only allowed peripheral cell binding. Euclidean distance maps to quantify the fluorescence signal from the bound ADC also showed that T-DM1 plus trastuzumab exhibited a more uniform distribution in fluorescence intensity, with T-DM1 alone showing a drop in signal towards the middle (**Figure 1B**). T-DM1 combined with pertuzumab showed a sharp decline in the ADC signal toward the center of the spheroid.

**Figure 1:**
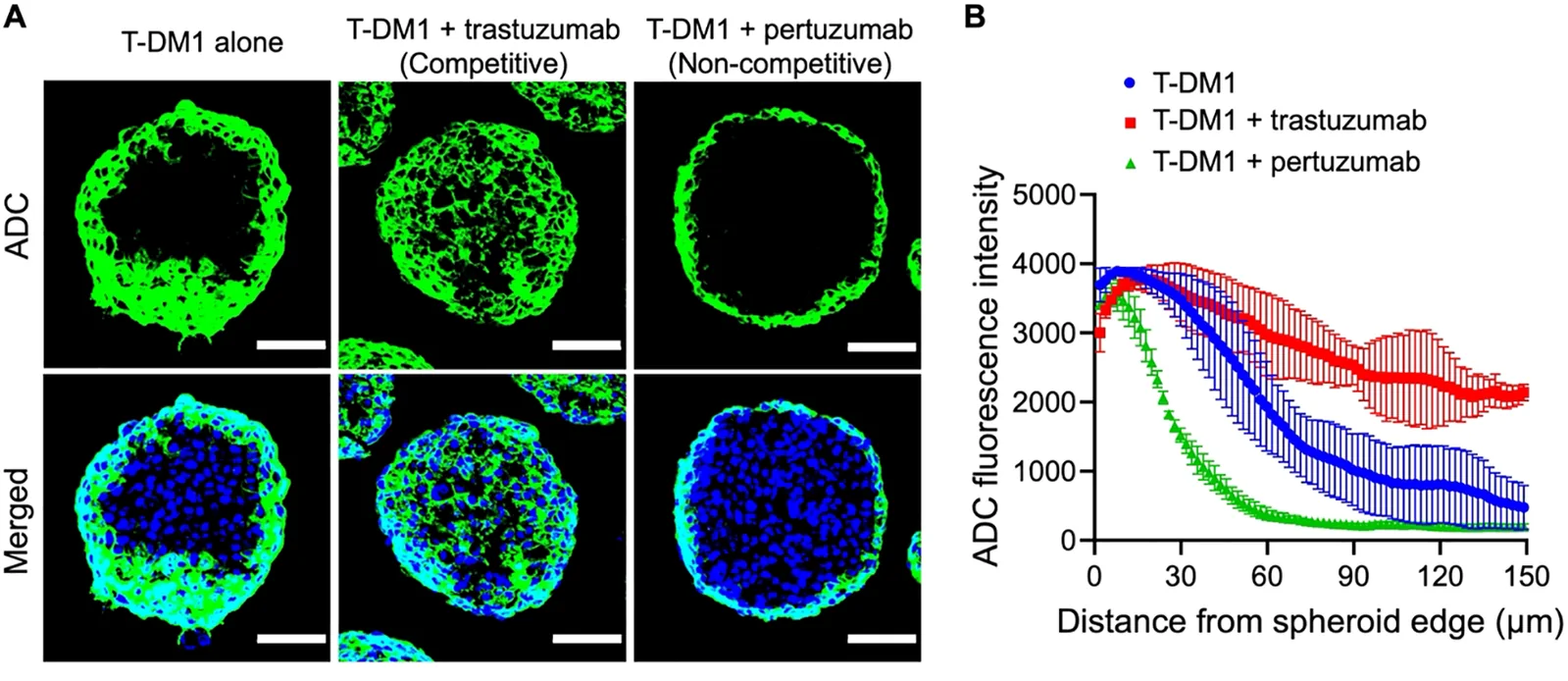
Immunofluorescence microscopy of T-DM1 alone or with trastuzumab or pertuzumab in HCC1954 spheroids. **(A)** The spheroids were treated with 300 nmol/L T-DM1-AF647 (green) with or without 900 nmol/L trastuzumab or pertuzumab. Cell nuclei were stained with Hoechst 33342 (blue). T-DM1-AF647 alone penetrated 2–3 cell layers (*left*) while co-incubation with trastuzumab competes with T-DM1 and enables T-DM1 to penetrate to the center (*middle*). Co-administration with pertuzumab reduces ADC penetration within the spheroids (*right*). **(B)** Euclidean distance map analysis of the spheroids, which reports the extent of ADC penetration (mean ± SD). Triplicates of immunofluorescence images were used for each group. Scale bar = 100 μm.

### Co-administration of trastuzumab with T-DXd improves penetration while pertuzumab leads to perivascular distribution *in vivo*

To examine whether ADC penetration shows similar trends in the presence of competitive and non-competitive antibodies *in vivo*, HER2+ gastric tumor xenografts were treated with fluorescently labeled T-DXd (trastuzumab deruxtecan). T-DXd is another HER2-targeting ADC with a bystander payload that has demonstrated greater clinical efficacy than T-DM1 (43, 44). A clinical dose of T-DXd (6.4 mg/kg) showed perivascular distribution in NCI-N87 tumor xenografts (**Figure 2**, *left*), similar to T-DM1 administered at 3.6 mg/kg (8). Co-administration of naked trastuzumab at a 1:3 ratio, 6.4 mg/kg of T-DXd and 19.2 mg/kg of trastuzumab, increased penetration and allowed the ADC to reach more cells (**Figure 2**, *middle*), consistent with literature reports showing that T-DXd and trastuzumab combinations improved penetration and efficacy (45). However, pertuzumab co-administered at the same 19.2 mg/kg dose did not affect the penetration of the ADC, exhibiting a similar heterogeneous distribution to T-DXd monotherapy (**Figure 2**, *right*).

**Figure 2:**
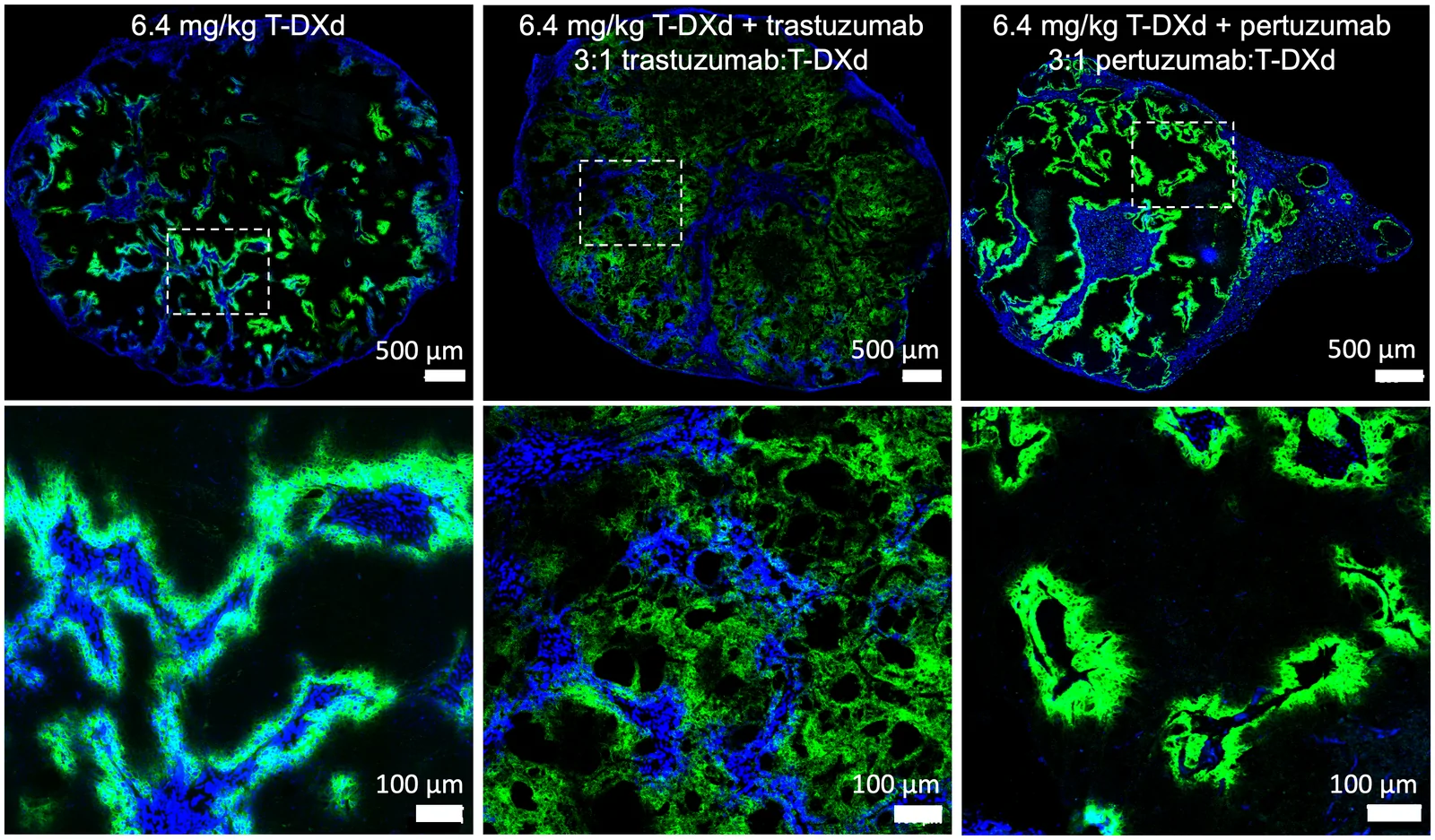
*In vivo* distribution of fluorescently labeled T-DXd alone or in combination with trastuzumab or pertuzumab. NCI-N87 xenografts (n = 3/group) were used to image ADC penetration *in vivo*. Once the tumor volume reached 250 mm^3^, 6.4 mg/kg of T-DXd conjugated to AF680 was administered through tail vein injection with or without unlabeled 19.2 mg/kg trastuzumab or pertuzumab. 15 mg/kg of Hoechst 33342 was administered before euthanasia to stain epithelial cells near functional blood vessels (blue). The tumors were resected 24 h later. T-DXd (green) alone exhibited a perivascular distribution (*left*), whereas the addition of a trastuzumab carrier antibody increased ADC penetration (*middle*) due to competitive binding. In contrast, the non-competitive pertuzumab did not enhance penetration (*right*).

These results are consistent with many studies showing improved responses when using a competitive carrier antibody to overcome the delivery challenge posed by high-affinity ADCs in high-expression tumors (8, 45–48). Based on these data, an ISAC may benefit from a competitive antibody to improve tissue penetration. However, this approach has limitations, including reduced payload delivery to low-expressing cells and/or heterogeneous expression in patients (31, 49). In a low-expression HER2 system, co-administration of carrier antibody has led to lower efficacy due to the decreased numbers of ADCs delivered to cells below a lethal level (31, 49). With heterogeneous HER2 expression (50), this poses challenges, including the selection of low-expressing cells or resistance to trastuzumab and pertuzumab (51). To help overcome these challenges, we previously generated a high-avidity, low-affinity (HALA) trastuzumab mutant, which is able to compete with ADC on high-expression cells but preserves ADC binding to low-expression cells (31, 38). The use of HALA antibodies could help the delivery challenge of ADCs across varying expression levels by improving penetration in high-expression tumors while maintaining payload concentration in low-expression cells. Additionally, the co-administration of an antibody enables greater Fc-effector function, which motivated us to utilize a HALA antibody with an enhanced Fc-binding domain, similar to margetuximab, a trastuzumab-based antibody with Fc-mutations that enhance *in vitro* ADCC responses (52). A ratio of 5:1 for the HALA antibody:ADC was shown to be optimal for enhanced efficacy *in vivo*.

### Co-administration of HALA antibody and pertuzumab with T-DXd leads to improved pharmacodynamics in 3D spheroids

We next demonstrated that T-DXd tissue penetration is similar to T-DM1 in tumor spheroids (**Figure 3**). To examine the tumor distribution and pharmacodynamics of ADC and antibody combinations, the same HCC1954 spheroid system in **Figure 1** was used in addition to γH2AX staining as a pharmacodynamic marker of DNA damage from the payload. A sub-saturating dose of T-DXd-AF647 was incubated with or without co-administered antibodies. Similar to T-DM1, a peripheral distribution (green) was observed with T-DXd incubation alone (**Figure 3A**). The pharmacodynamic staining (red) exhibited moderate intensity towards the center at both 48 and 72 h (**Figure 3B**), indicating a DNA damage associated with bystander killing effect of the DXd payload. With competitive antibody co-administration, the ADC was distributed more homogeneously due to increased penetration resulting from binding competition with the co-administered eFc-HALA antibody, and the pharmacodynamic signal was similar to that of T-DXd-treated spheroids. As expected, co-administration of pertuzumab did not improve ADC penetration. However, it did exhibit a stronger pharmacodynamic signal at 48 and 72 h compared to T-DXd or T-DXd plus eFc-HALA antibody-treated spheroids, indicating that faster internalization may increase intracellular payload delivery. Additionally, the shape of spheroids began to deform more rapidly due to peripheral cell death.

**Figure 3:**
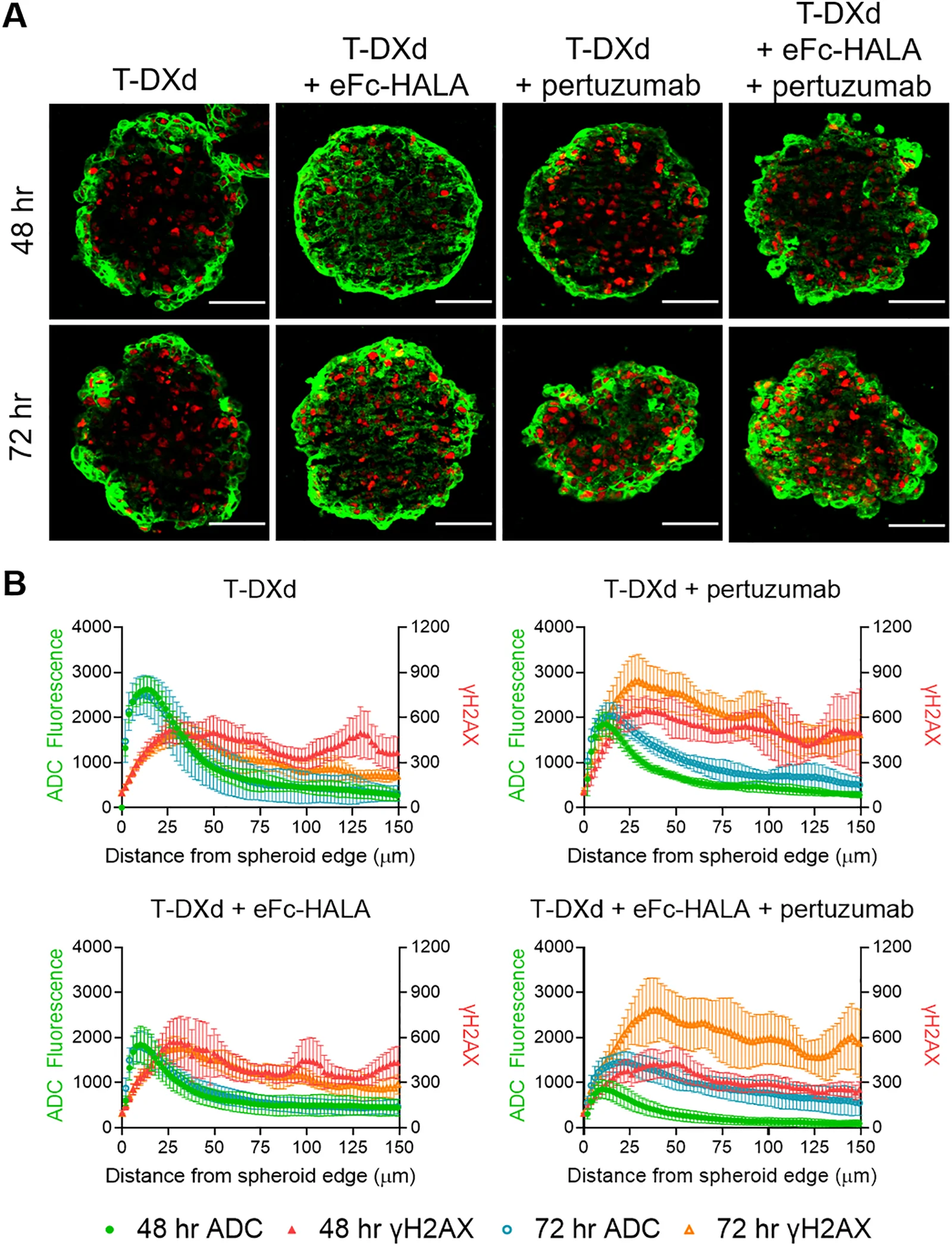
Immunofluorescence microscopy of T-DXd distribution and payload response in spheroids with or without combination treatment. **(A)** Spheroids were treated with 200 nmol/L T-DXd-AF647 (green) and 1000 nmol/L of 1) eFc-HALA antibody, 2) pertuzumab, or 3) both. Immunofluorescence staining of γH2AX-PE (red) showed DNA damage as the pharmacodynamic signal of payload response. **(B)** Euclidean distance maps report ADC fluorescence in green for 48 h and cobalt for 72 h (mean ± SD). Pharmacodynamic signals are shown in red for 48 h and orange for 72 h. While the eFc-HALA antibody increased ADC penetration, the pharmacodynamic signal was higher with pertuzumab co-incubation, especially at 72 h. Seven images were used in Euclidean distance maps, except for the 48-hour T-DXd, which used only five images. Scale bar = 100 μm.

Based on the high pharmacodynamic signal with pertuzumab co-administration, we investigated the possibility of achieving both faster internalization and increased penetration with multiple antibodies. Notably, the triple combination of T-DXd, eFc-HALA antibody, and pertuzumab showed the greatest pharmacodynamic signal at 72 h, with the center of spheroids also exhibiting a higher signal compared to all other conditions (**Figure 3**). These results highlighted the potential to achieve both improved distribution and increased cellular internalization with a combination of competitive and non-competitive antibodies. Therefore, a triple combination strategy could outperform the double combination therapy by increasing both tissue-level (tumor distribution) and cellular-level (internalization) kinetics.

### Epitope selection between ADC and ISAC impacts the relative delivery of cytotoxic and immunostimulatory payloads to cancer cells versus immune cells

Aside from the cytotoxic ADC distribution and efficacy, Fc-mediated delivery to immune cells is an important mechanism for ISAC payload delivery. Antibody, ADC and ISAC combinations can impact immune cell (Fc-mediated) internalization relative to HER2 (target-mediated) internalization. To quantify the cellular uptake rates of ADCs and ISACs in cancer and immune cells in a co-administration setting, a co-culture cellular uptake assay was performed using human HER2-expressing murine breast cancer cells (E0771-HER2) and mouse macrophage-like cells (J774A.1). A fluorescent dye, Alexa Fluor 488, was used as a payload surrogate to avoid confounding effects of cytotoxic or immune-stimulating payloads. After incubation, any non-internalized fluorophore remaining on the cell surface was quenched using Trypan Blue to measure intracellular delivery. The ISAC-surrogates were incubated at a 3-fold higher concentration than the ADC-surrogate. The fluorescence signal normalized to the degree of labeling represents the relative amount of internalized ADC- or ISAC-surrogates in cancer and immune cells.

The uptake of the trastuzumab ADC-surrogate (i.e., AF488 conjugated trastuzumab in **Figure 4A**) was similar in cancer cells and macrophages when incubated alone at a saturating concentration over the 4 h incubation time. This is consistent with the high Fc receptor concentration on J774A.1 cells (53), the moderate HER2 expression on E0771-HER2 cells [~250,000 HER2/cell (31)], and the faster internalization of FcγRI than HER2 (54–57). When a trastuzumab carrier antibody targeting the same (competitive) epitope was co-incubated, the internalized ADC intensity decreased significantly in both cell types, as the non-fluorescent antibody partially blocks the binding sites for the ADC and thereby reduces its uptake. In contrast, the internalized ADC signal in cancer cells was numerically higher when pertuzumab was present (non-competitive epitope), consistent with crosslinking the receptor and driving faster internalization, as seen with the higher spheroid pharmacodynamic signal when the crosslinking antibody was added. However, this finding was not statistically significant over the 4 h incubation time (*p* = 0.0558). The macrophage uptake of the ADC in the presence of pertuzumab was similar to that of ADC alone (*p* = 0.3784), indicating that antibody crosslinking of HER2 did not significantly reduce Fc-mediated internalization.

**Figure 4:**
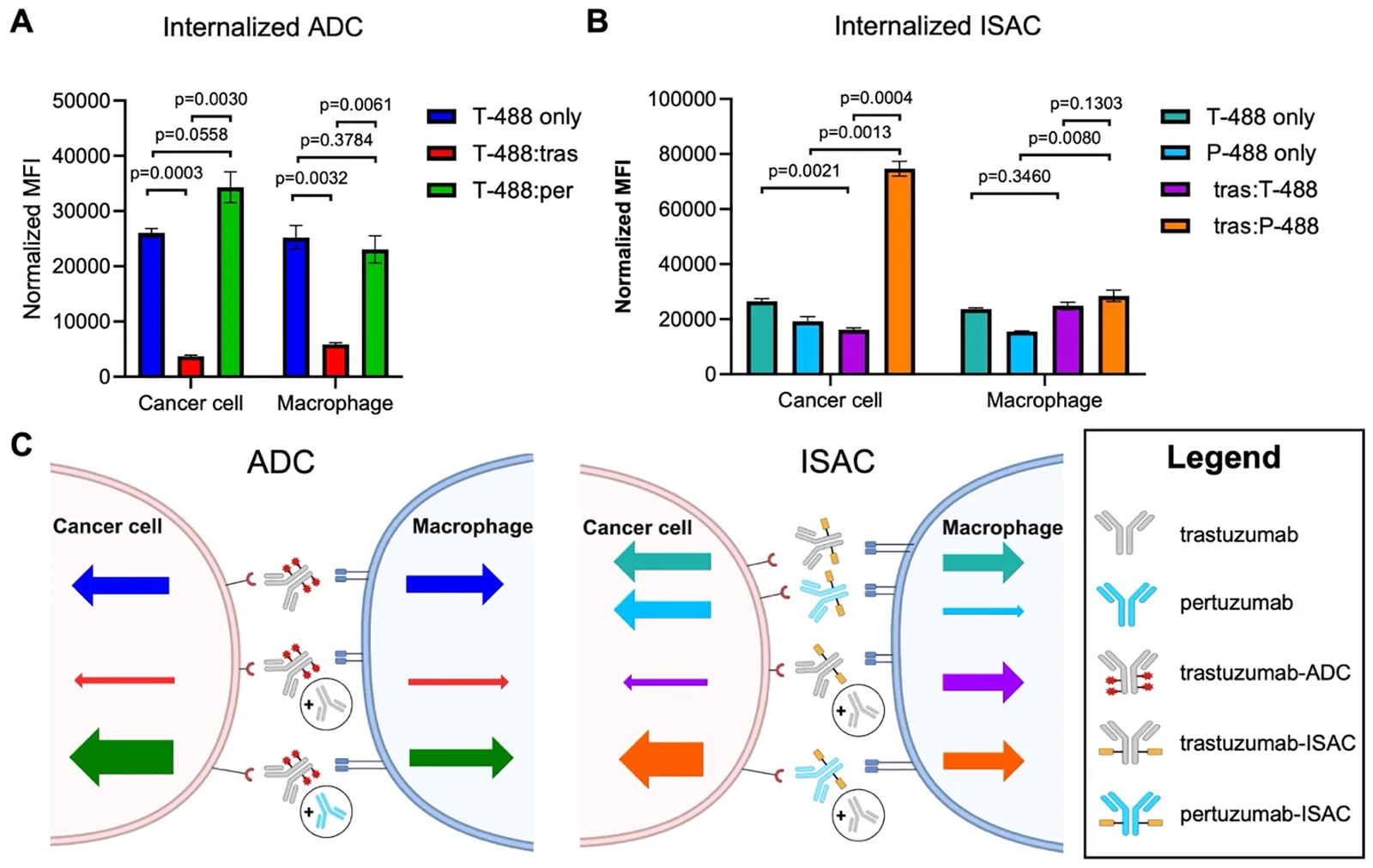
Co-culture assay with cancer and immune cells for quantifying internalization of ADC and ISAC surrogates during co-administration. **(A)** E0771-HER2 and J774A.1 cells were co-cultured in a 96-well plate. ADC surrogate (i.e., T-488; 40 nM) was incubated with 120 nM of unconjugated trastuzumab or pertuzumab to quantify the fluorescence intensity of internalized ADC when carrier antibody is present. The trastuzumab-ADC internalizes numerically more in cancer cells when pertuzumab is present (p = 0.0558 for T-488 only versus T-488:per) versus the lower internalization when additional trastuzumab-carrier antibody is present and competes for binding (p = 0.0003 and 0.0032 for T-488 only versus T-488:tras in cancer cells and macrophages, **(B)** In the same co-culture setting, ISAC surrogates (i.e., T-488 and P-488; 120 nM) were incubated with 40 nM of naked trastuzumab (ADC surrogate) to quantify levels of internalized ISAC. The pertuzumab-ISAC has higher cancer cell uptake due to crosslinking with trastuzumab, but both trastuzumab- and pertuzumab-ISACs have similar macrophage uptake (*p* = 0.0004 and 0.13 for cancer cells and macrophages, respectively; Mean ± SD). **(C)** Schematic of internalized ADC and ISAC when either competitive or non-competitive antibodies were present. Overall, non-competitive, crosslinking combinations increased ISAC uptake into cancer cells and produced a numerical increase in ADC uptake that was not statistically significant, while having minimal impact on macrophage uptake. The mean fluorescence intensity (MFI) was normalized to the degree of labeling of the fluorescent dye for comparison. Created with BioRender.com.

Similarly, the pertuzumab ISAC-surrogate (i.e., AF488-conjugated pertuzumab in **Figure 4B**) showed significantly higher uptake in cancer cells when the crosslinking antibody (non-competitive epitope) was present, driving substantially higher cancer cell uptake of the ISAC (Tras:P-488). Interestingly, pertuzumab uptake was approximately four-fold higher in the presence of trastuzumab compared to pertuzumab alone, potentially due to the pH-dependent binding of pertuzumab to HER2 and/or trafficking differences (58). Pertuzumab can dissociate more readily in the endosome, whereas trastuzumab can recycle back to the cell surface. Thus, the crosslinking of trastuzumab and pertuzumab promotes receptor clustering and internalization of both antibodies. Afterward, trastuzumab appears to recycle, while pertuzumab dissociates intracellularly. In contrast, with competitive binding, the cancer cell internalized much less trastuzumab-ISAC (*p* = 0.0021), but the macrophage uptake was similar between the two groups (*p* = 0.3460).

In summary, the combination of non-competitive, crosslinking antibodies showed a numerical increase in ADC uptake into cancer cells, although this difference did not reach statistical significance, while significantly increasing ISAC uptake. The impact on macrophage uptake was minimal. In contrast, the combination of competitive antibodies lowers the ADC uptake into cancer cells and immune cells, which can improve distribution, and lowers ISAC uptake into cancer cells while maintaining ISAC immune cell uptake, shifting the balance towards immune cells.

The co-culture assays indicated that a competitive antibody may be more suitable for a TLR7-ISAC by enhancing the cytotoxic ADC tissue distribution, reducing cytotoxic delivery to immune cells, and decreasing TLR7 agonist ISAC uptake into cancer cells that are not expected to be the primary target of TLR7 agonism, while maintaining ISAC immune cell uptake. To evaluate this *in vivo*, we first tested the efficacy of TLR7 agonist ISAC monotherapy. A moderate potency TLR7 agonist was selected to enable higher antibody dosing of the ISAC, which leverages the antibody mechanisms of action, including the Fc-effector functions of trastuzumab and the signaling blockade of pertuzumab (59, 60). This potency is lower than that of NJH-395 to avoid toxicity, but higher than BDC-1001 (which was tolerated clinically at 20 mg/kg biweekly), with a similar strategy of reducing potency to increase dosing, analogous to topoisomerase payloads versus DNA alkylators for cytotoxic payloads (**Supplementary Figure S1**) (28). We conjugated a commercial TLR7 agonist to eFc-HALA antibody and pertuzumab to compare performance *in vitro* and *in vivo*.

To select the DAR on the TLR7 ISAC, we evaluated DAR2 and DAR8 TLR7 ISACs using an antibody that cross-reacts with both human and mouse antigens, as well as a cytotoxic payload with potency in both human and mouse cells in a syngeneic mouse model, to better capture potential toxic effects (**Supplementary Figure S2**). While the ADC alone resulted in negligible weight loss, significant toxicity was observed when combined with the TLR7 agonist at DAR8. In contrast, the ADC combined with DAR2 TLR7 ISAC had tolerable and transient weight loss. The ADC payload dose was ~10-fold higher than the clinically tolerated amount, suggesting that a lower, clinically tolerated cytotoxic payload dose may permit higher ISAC dosing. Also, because our combination strategy was designed for cytotoxic ADC to drive tumor regression while the ISAC provided adjunctive immune stimulation and potentially enhanced response durability, we moved forward with a low DAR ISAC (DAR2). Unlike PBD, the DXd payload has low-to-negligible potency against mouse tumor cells (15); therefore, we utilized athymic nude mice that maintain innate immune cells to test the efficacy of ADC and ISAC combinations.

To evaluate *in vitro* potency, eFc-HALA-TLR7, pertuzumab-TLR7, trastuzumab, pertuzumab, and eFc-HALA were incubated with HEK-Blue mTLR7 cells. Both ISACs induced similar NF-κB activating responses (**Figure 5A**), with eFc-HALA-TLR7 resulting in a 5.2-fold increase and pertuzumab-TLR7 a 5.7-fold increase. In contrast, the unconjugated antibodies showed no activation. We also tested the impact of antibody combinations on an ADCC reporter cell line (**Figure 5B**). When normalized to the trastuzumab concentration, the addition of pertuzumab slightly decreased the maximum activation with little impact at lower concentrations. This is consistent with moderate interactions between antibody combinations on the cell surface (61). Combining the eFc-HALA antibody with trastuzumab slightly increased the maximum activation and increased activity at lower concentrations. Overall, the combination of trastuzumab, pertuzumab, and eFc-HALA antibody had higher activity at low concentrations but a similar maximum induction as the clinical combination of trastuzumab plus pertuzumab.

**Figure 5:**
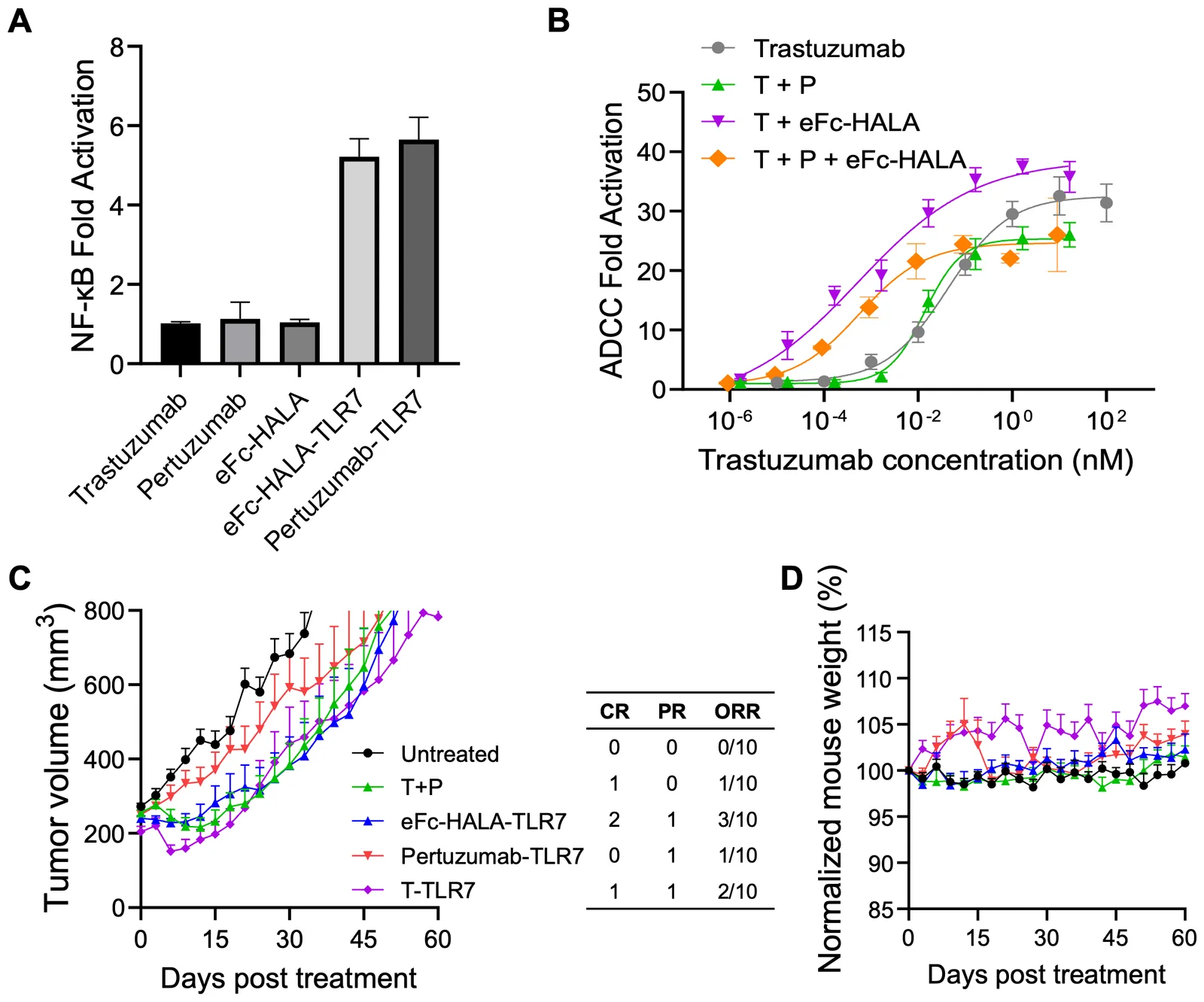
*In vitro* reporter assays and *in vivo* efficacy of ISACs. **(A)** HEK-Blue mTLR7 reporter cells were incubated with 100 nM of trastuzumab (T), pertuzumab (P), eFc-HALA antibody, eFc-HALA-TLR7, and pertuzumab-TLR7. Based on the reporter assay, both ISACs showed similar TLR7 activation *in vitro* at the same concentration. The control antibodies showed no induction. **(B)** ADCC reporter cells co-cultured with NCI-N87 cells were incubated with different antibody combinations at fixed ratios relative to trastuzumab (5:1). The addition of pertuzumab slightly decreased the maximum fold activation, while eFc-HALA antibody increased response compared to trastuzumab alone. The triple combination of antibodies showed slightly lower maximum ADCC activation at high concentrations, but higher activation at lower concentrations. **(C)** To test the efficacy *in vivo*, NCI-N87 bearing nude mice received 1) no treatment, 2) 6.4 mg/kg trastuzumab and 32 mg/kg pertuzumab, 3) 32 mg/kg of eFc-HALA-TLR7, 4) 32 mg/kg of pertuzumab-TLR7, or 5) 32 mg/kg trastuzumab-TLR7 (n = 10/group; n = 8 for untreated). Trastuzumab and pertuzumab treatment induced moderate tumor regression, indicating that growth signaling blockade benefits the efficacy. The eFc-HALA-TLR7 cohort had similar tumor regression to the combination therapy, whereas pertuzumab-TLR7 was the least effective, with some growth inhibition but without regression. The number of complete responses (CR) with no detectable tumor, partial responses (PR) with > 50% reduction in tumor volume, and overall response rate (ORR) calculated as the total CRs plus PRs versus number of animals treated were tabulated. **(D)** All mice that received treatment did not show significant weight loss, indicating the treatments were tolerable even at high doses. In **(A, B)**, the *in vitro* assay data represent mean ± SD. In **(C, D)**, the *in vivo* efficacy data represent mean ± SEM.

### The eFc-HALA-TLR7 ISAC is more efficacious than pertuzumab-TLR7 in HER2+ gastric cancer xenograft

To test the efficacy of the different ISACs *in vivo*, the ISACs or trastuzumab and pertuzumab antibodies were administered through a single tail vein injection in NCI-N87 bearing nude mice once the tumor volume reached 250 mm^3^. Pertuzumab-TLR7 had a slight impact on tumor growth inhibition, with one partial responder, but was less efficacious than T-TLR7 or eFc-HALA-TLR7 treatment, which resulted in one partial response and one complete responder, or one partial responder and two complete responders, respectively. While this motivates the use of trastuzumab, pertuzumab has the potential to reduce cell signaling in combination with trastuzumab (59). Therefore, we tested the combination of trastuzumab plus pertuzumab to quantify the magnitude of cell signaling blockade relative to TLR7 agonism. Trastuzumab and pertuzumab at a 1:5 ratio exhibited moderate tumor growth inhibition, similar to eFc-HALA-TLR7 monotherapy, but only one complete responder (**Figure 5C**). None of the treatments showed significant weight loss, indicating tolerability of the ISACs at these doses (**Figure 5D**). Together, these data indicated that eFc-HALA-TLR7 was the more effective ISAC, but neither ISAC monotherapy nor trastuzumab and pertuzumab combination treatment effectively reduced the tumor volumes. Additionally, despite the lower efficacy of the pertuzumab ISAC, the impact of pertuzumab cross-linking on cell signaling blockade provided an additional benefit. Therefore, we next tested T-DXd ADC and eFc-HALA-TLR7 or pertuzumab-TLR7 ISAC combination therapy *in vivo*.

### Triple combination of ADC, ISAC, and crosslinking antibody demonstrates the strongest efficacy

In NCI-N87 cancer xenografts, the efficacy was evaluated for different combination treatments with T-DXd: a competitive eFc-HALA-TLR7 ISAC, a non-competitive pertuzumab-TLR7 ISAC, and unconjugated pertuzumab. T-DXd generates strong efficacy in the NCI-N87 tumor model (**Figure 6A**), providing a high bar for improvement. T-DXd combined with pertuzumab, eFc-HALA-TLR7 ISAC, or pertuzumab-TLR7 ISAC showed greater tumor reduction and a higher overall response rate than T-DXd alone (**Figure 6A**). This highlights the potential benefit of improved HER2 signaling blockade by combining the trastuzumab ADC with pertuzumab or the pertuzumab ISAC despite the reduced tissue penetration of the ADC. Bystander effects of the payload help partially offset the limited tissue penetration observed *in vivo* (**Supplementary Figure S3**). The improved tissue penetration by combining the ADC with a competitive ISAC with increased ADCC responses can also benefit efficacy due to more efficient payload delivery and immune response. Notably, the triple combination of T-DXd, eFc-HALA-TLR7, and pertuzumab achieved the highest tumor reduction, demonstrating statistical significance compared to T-DXd monotherapy at 90 days post-treatment (**Figure 6B**, **Supplementary Table S3**).

**Figure 6:**
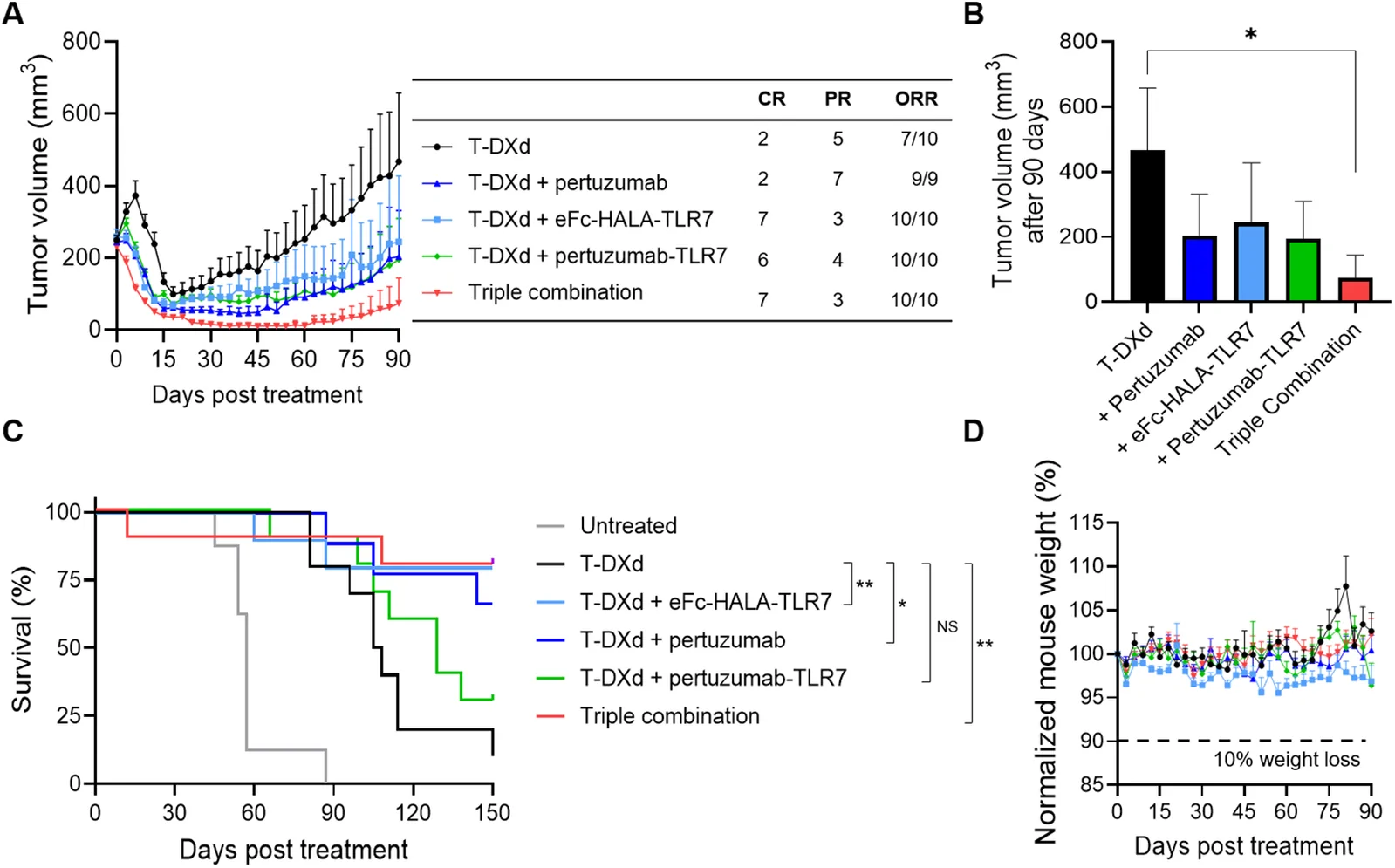
Efficacy of T-DXd and ISAC combination therapy in NCI-N87 xenografts. **(A)** *In vivo* effect of T-DXd (6.4 mg/kg) alone or co-administered with pertuzumab, eFc-HALA-TLR7, pertuzumab-TLR7 or eFc-HALA-TLR7 and pertuzumab (32 mg/kg of each) in nude mice bearing NCI-N87 tumors (n = 10/group; n = 9 for T-DXd + Pertuzumab) (Mean ± SEM). Treatments containing a TLR7 agonist increased the number of complete responses. **(B)** Tumor volume comparison 90 days after the treatments. T-DXd compared to triple combination showed statistical significance (*p* = 0.0158). **(C)** Kaplan-Meier survival curve shows improved survival for combination therapy versus T-DXd alone (T-DXd + eFc-HALA-TLR7, *p* = 0.0065; T-DXd + pertuzumab, *p =* 0.0156; T-DXd + pertuzumab-TLR7, *p* = 0.2165; Triple combination, *p* = 0.0036). Death or euthanasia was considered a survival event; animals alive at Day 150 were censored. Notably, several mice experienced death or euthanasia prior to 150 days despite tumor volumes not reaching humane endpoints (**Supplementary Table S2**). **(D)** Mouse weight loss compared to the initial weight was not significant after 90 days of treatment, providing evidence of tolerability during the measured period. *p=< 0.05, **p= < 0.01.

Interestingly, while T-DXd combined with either pertuzumab or pertuzumab-TLR7 demonstrated a 100% overall response rate, T-DXd combined with pertuzumab-TLR7 showed a higher number of complete responses (60% vs. 22%), indicating the contribution of TLR7 agonism in the depth of response. In fact, all three treatments with a TLR7 agonist combined with T-DXd resulted in high numbers of complete responses (60-70%) compared to T-DXd alone (20%). These strong responses resulted in prolonged survival times, and the mice were monitored for 150 days (**Figure 6C**). Several mice experienced death or required euthanasia due to health concerns prior to 150 days or tumors reaching the humane endpoint (**Supplementary Table S2**), resulting in T-DXd plus eFc-HALA-TLR7 or the triple combination having the highest number of surviving mice. In addition, the body weight changes were comparable across all treatment groups (**Figure 6D**) with less than 5% weight loss for all cohorts out to 150 days, suggesting acceptable tolerability for the combinations. However, caution should be exercised when interpreting the body weight data, as mice exhibit greater tolerability to the DXd payload, and the ADC does not bind to mouse HER2. This result indicates that the combination of faster internalization and penetration can effectively deliver both ADC and ISAC in solid tumors, consistent with the 3D spheroid results (**Figure 3**).

## Discussion

With increasing numbers of approved ADCs in the clinic, extensive efforts have been made to find combination therapies to leverage the multiple mechanisms of action of ADCs. One approach to treat immunologically ‘cold’ tumors unresponsive to checkpoint inhibition is the use of immune-stimulating payloads, or ISACs, which deliver immune-activating payloads to elicit a potent antitumor immune response. Our previous work has demonstrated the impact of utilizing competitive versus non-competitive carrier antibodies on ADC efficacy (8, 32, 33) with competitive antibodies improving penetration and efficacy, and non-competitive antibodies exhibiting contrasting outcomes depending on saturating versus sub-saturating doses. Several ISACs targeting different HER2 epitopes have been generated and tested in the clinic (62–64), and here we demonstrated how the epitope selection impacts efficacy of ADC and ISAC combinations.

We first demonstrated the impact of competitive and non-competitive antibodies on ADC distribution in 3D spheroids and human cancer xenografts. At sub-saturating concentrations, T-DM1 alone showed peripheral distribution in tumor spheroids due to rapid binding, but it was able to reach the center of spheroids when co-incubated with trastuzumab (**Figure 1**). Trastuzumab partially blocks the binding sites of the ADC due to the shared epitope, enabling the ADC to diffuse deeper into the tissue. The opposite occurs with the non-competitive antibody pertuzumab, where the penetration was reduced due to increased ADC internalization from trastuzumab and pertuzumab crosslinking (37). This reduced distribution of T-DM1 when combined with pertuzumab could be a potential cause of the insufficient improvement in efficacy seen in clinical trials with T-DM1 and pertuzumab combination (65, 66) despite the benefit of HER2 signaling blockade. *In vivo*, similar trends were observed with antibody and ADC combinations, where the co-administration of T-DXd with trastuzumab increased the ADC tissue penetration, but pertuzumab co-administration with T-DXd showed perivascular distribution in HER2+ gastric cancer xenografts (**Figure 2**). Interestingly, although the T-DXd penetration was limited when a crosslinking antibody was added, we observed increased payload delivery in 3D tumor spheroids, likely from improved cellular internalization/delivery (**Figure 3**). T-DXd has a bystander payload that can diffuse into adjacent cells, partially offsetting some of the drawbacks from limited penetration, even though bystander killing is often less efficient than direct targeted cell killing (**Supplementary Figure S3**) (67). Strikingly, the triple combination involving T-DXd, eFc-HALA antibody, and pertuzumab exhibited both high ADC tissue penetration and high pharmacodynamic signal throughout the entire spheroid section, indicating that the use of competitive and non-competitive antibodies together could enhance the ADC and payload delivery simultaneously. Although driving faster cellular internalization to improve efficacy is context-dependent, as it may reduce the penetration (33), these results suggest a potential strategy for combination treatment to maximize efficacy with potent ADCs. Thinking of payload delivery as ‘resistances in series,’ where improving only one barrier results in the other barrier limiting efficacy, the competitive antibody helps overcome the first diffusion barrier in the tissue, while the crosslinking antibody overcomes the second cellular internalization barrier.

The contrasting effects of combining cytotoxic ADCs with competitive and non-competitive antibodies raised the question of which antibody should be used for a TLR7-ISAC. TLR7 agonists can provide additional antitumor activity by inducing proinflammatory cytokines and type I interferons (IFNs) in various immune cells, including macrophages, natural killer (NK) cells, and dendritic cells (DCs) (68). Similar to ADCs, a majority of the ISAC is not delivered to the tumor and can be taken up non-specifically by healthy cells, such as B-cells, which can drive ADA responses (29). Therefore, a moderate potency TLR7 agonist payload was selected to reduce on-target off-tumor toxicity while mediating a productive immune response. The rationale for combination therapy is similar to the ADC and ICI combinations that have shown success for tumors responsive to ICI (18). ADCs invoke immunogenic cell death while sparing beneficial immune cells activated by immunotherapy. By pairing ISACs with ADCs, the ADC can drive tumor regression, while the ISAC enhances the immune response to increase durability and complete responses, particularly in less immunogenic tumors where checkpoint inhibition is less effective. The combination also reduces the need for aggressive immune activation and single-agent activity from the ISAC alone, potentially increasing the therapeutic window.

For the competing epitope antibody, we utilized an engineered antibody with mutations in the Fc domain to enhance activating effector function (**Figure 5**) and reduce HER2 affinity, denoted as eFc-HALA antibody (38). The low affinity and high avidity of eFc-HALA antibody for HER2 enables competition with the ADC for binding on high HER2-expressing cells to promote increased tumor tissue penetration but does not compete for binding on low HER2-expressing cells, allowing only the ADC to internalize and deliver sufficient toxic payload (31). Because the trastuzumab and pertuzumab combination showed growth inhibition in a preclinical model (59), we also tested pertuzumab with or without a TLR7 agonist to leverage HER2 cell-signaling inhibition as another mechanism of action alongside TLR7 agonism with this non-competitive antibody in order to select the optimal ISAC for ADC combination therapy.

A single 32 mg/kg dose of eFc-HALA-TLR7 showed moderate tumor regression and growth delay, similar in potency to the clinically validated trastuzumab and pertuzumab combination (**Figure 5**). This level of efficacy is not observed with trastuzumab itself (e.g., three doses of 20 mg/kg of trastuzumab) (59). The use of a moderate potency payload DAR2 ISAC also resulted in negligible weight loss in this model. In comparison, pertuzumab-TLR7, which showed similar *in vitro* NF-κB activation as eFc-HALA-TLR7, slowed tumor growth but was not as efficacious as trastuzumab plus pertuzumab or eFc-HALA-TLR7 treatment and resulted in only a single partial response. This difference highlights that TLR7 agonist activity alone does not determine the efficacy of an ISAC. In addition to TLR7 agonist potency, the antibody format and dosing can influence HER2 receptor engagement and internalization, Fc-mediated uptake by immune cells, and tissue distribution. Therefore, differences in antibody-mediated trafficking and cellular interactions contribute to the distinct *in vivo* activities observed for the two ISACs despite their similar TLR7 reporter responses. However, none of these agents achieved substantial tumor volume reduction or high overall response rates. In large, immunosuppressive tumors, it may be challenging for ISACs alone to elicit robust antitumor immunity without risking on-target, off-tumor toxicity (29). This underscores the advantage of incorporating cytotoxic ADCs to achieve tumor growth control, creating a tumor microenvironment in which ISACs can amplify immune activation without substantial toxicity. In this context, T-DXd monotherapy at the clinical dose resulted in substantial tumor regression, consistent with the efficacy in this xenograft model. However, only two complete responses were achieved by the ADC alone.

The addition of eFc-HALA-TLR7 further decreased the tumor size, resulting in a statistically significant improvement in survival, and 7 out of 10 mice achieved complete responses. Co-administration of either pertuzumab or pertuzumab-TLR7 also produced comparable tumor regression. Importantly, complete responses were more frequent when the TLR7 agonist was included even at doses that did not regress tumors with monotherapy. In these immunodeficient mice, T-DXd combined with eFc-HALA-TLR7 or pertuzumab-TLR7 resulted in 7 and 6 complete responses, respectively, compared to only 2 or 0 complete responses for T-DXd plus pertuzumab or with eFc-HALA. The lack of complete responses across our study and a previously published study with T-DXd and HALA (8/8 PR, 0/8 CR) (31) suggests the addition of TLR7 agonism may increase the depth of response and enable complete responses. This is consistent with the ADC driving tumor regression and the ISAC improving the durability of response. Notably, a triple combination of T-DXd, eFc-HALA-TLR7, and pertuzumab resulted in the greatest efficacy and a large number of 7 complete responses, with a statistically significant decrease in tumor size, indicating that the combination of cytotoxic ADC in addition to TLR7 agonists was needed to significantly reduce the tumor size (**Figure 6**). Similar to single-agent therapy, the combination of ADCs and ISACs did not demonstrate significant weight loss, indicating that the treatments were tolerable in this preclinical model.

Clinically, there are currently far fewer trials utilizing ISACs than cytotoxic ADCs, and clinical data on ISAC and ADC combinations are even more limited. A single patient was treated with a combination of trastuzumab deruxtecan with pertuzumab zuvotolimod (SBT-6050) (69). The patient exhibited a partial response but also cytokine release syndrome, which was prevalent with the potent TLR8 agonist monotherapy (NCT05091528). No patients were tested with trastuzumab imbotolimod (BDC-1001) and trastuzumab deruxtecan (T-DXd) combinations. Antibody combinations, including checkpoint inhibitors, have been tested in more patients, including trastuzumab imbotolimod (BDC-1001) with pertuzumab in patients previously treated with T-DXd (NCT05954143). The results of this trial have not been published, however, and there are no reports of concurrent treatment with the ISAC and T-DXd.

Given the complexity of combination therapy, these studies have several limitations. First, the athymic nude mice used in the study retain innate immune cells that can respond to TLR7 activation but lack functional T cells, limiting the ability to assess T-cell-mediated adaptive antitumor immunity or immune memory. Therefore, the increased complete response rates and prolonged survival observed here are driven by the cytotoxic ADC, innate immune effects of the ISAC, and HER2 signaling blockade in the context of competitive and non-competitive targeting strategies. Studies in immunocompetent models with appropriate cross-reactive reagents are needed to establish the contribution of adaptive immunity to the durability of these responses, which are currently limited by the low sensitivity of mouse cancer cells to T-DXd. Second, only a TLR7 agonist was studied for the ISAC payload (62). TLRs are primarily expressed in immune cells (70), consequently increasing uptake in cancer cells by combining non-competitive antibodies is unlikely to provide significant advantages compared to increasing uptake in immune cells (**Figure 4**). However, the optimal combination is likely dependent on the specific ISAC pathway and cancer receptor expression. For instance, BDC-1001 (Bolt Biotherapeutics) and XMT-2056 (Servier) are HER2-targeting ISACs that have been evaluated in combination with non-competitive antibodies, with BDC-1001 having been clinically tested and XMT-2056 assessed preclinically *in vivo* (62, 71). BDC-1001 utilizes a TLR7/8 agonist, which may not yield substantial benefits from increased cancer cell uptake, except in tumors that express these receptors (72). In contrast, XMT-2056 employs a STING agonist that can activate the cGAS-STING pathway and inflammatory responses in both cancer cells, where it is typically retained (73), and immune cells (74). Thus, non-competitive ISACs with ADC combination may be desired if utilizing STING agonists. Aside from the immune-stimulating pathway, the pharmacokinetic properties of the ISAC payloads can be modified to increase or decrease bystander cell uptake. For example, XMT-2056 limits bystander uptake to avoid STING-mediated T-cell suppression (71). The TLR7 agonist employed here is commercially available, to facilitate benchmarking studies, and utilizes a non-cleavable linker, thereby limiting bystander effects but potentially increasing toxicity (75). However, alternative strategies to use cancer cell uptake and release of bystander ISAC payloads have also been developed (76). Likewise, we focused on T-DXd as the model ADC. The ADC payload can also have differential effects on immune cells. Some payloads, like topoisomerase inhibitors and microtubule inhibitors, can activate immune cells (15, 16, 23, 24) while others, such as calicheamicins, can kill immune cells (14, 77, 78). Protein engineering of the Fc domain or glycosylation can be used to alter Fc-mediated uptake for either compound, providing another layer of control.

Two related approaches include (i) targeting independent antigens to deliver the separate payloads (e.g. combining TAK-500 targeting immune cells with a separate ADC) (79) or (ii) one antibody with dual payloads (e.g. B7-H3, TROP2, HER2) (75, 76, 80–82). Each has pros and cons depending on the target(s) and payloads. Targeting separate antigens requires co-expression of the targets within the tumor to deliver both payloads. Unlike a bispecific ‘AND’ gate, expression of one of the targets in normal tissue will still deliver one of the payloads but not both. It also requires use of two separate agents, which is more feasible from a commercial and regulatory standpoint when one of the ADCs is already FDA-approved. Targeting separate antigens retains the ability to dose-reduce only one of the payloads due to toxicity, enabling tailoring to specific patients as an advantage. For (ii), dual payload approaches require a fixed ratio of the payloads, so separate dose reduction is not possible. It is also difficult to engineer differential payload delivery to cancer versus immune cell types. However, a strength is the simplified and broader patient selection by focusing on expression of a single target. Likewise, this can be better suited for novel targets where co-development of separate agents would yield significant regulatory hurdles and development costs. Despite the challenges associated with all these strategies, combination therapy has consistently led to better outcomes over the history of cancer treatment.

In conclusion, we have demonstrated that ADC combination therapy, utilizing multiple mechanisms of action, can increase tumor regression. Furthermore, the addition of a TLR7 ISAC enhances the number of durable complete responses. Our findings suggest that achieving both high tumor tissue penetration and rapid internalization is feasible and important in maximizing antitumor activities with ADCs and antibody/ISAC combinations. ADCs/ISACs/antibody combinations with competing epitopes increase tissue penetration while reducing cancer cell uptake, whereas non-competitive agents crosslink receptors, increasing cellular uptake while reducing penetration. For ISAC monotherapy, utilizing a trastuzumab antibody with enhanced Fc effector function for conjugation with a TLR7 agonist showed the strongest response. However, for combination therapy, the addition of pertuzumab to the ADC and ISAC combination resulted in the greatest efficacy, achieving maximum tissue penetration of the ADC through competitive binding with the ISAC and maximum cellular internalization and cell signaling blockade with the crosslinking of pertuzumab. Overall, this provides guidance for the rational selection of epitopes in ADC and ISAC combination therapy.

## Data Availability

The original contributions presented in the study are included in the article/**Supplementary Material**. Further inquiries can be directed to the corresponding authors.
