# Supplementary material for: Epitope selection for combination therapy using antibody-drug conjugates and immune immune-stimulating antibody conjugates
Source: Front Immunol. 2026 Oct 8;17:1960790. doi: 10.3389/fimmu.2026.1960790 (PMC13687061; doi:10.3389/fimmu.2026.1960790)
Supplement: Supplementary file 1 [file Table1.docx]

**Supplemental Table 1**

*In vivo* ISAC efficacy study design

| **Treatment group** | **Number of mice per group** | **Event Time and Details** |
| --- | --- | --- |
| Untreated | n = 8 | - 8 mice euthanized due to tumor size humane endpoint (D+48, 54, 57, 87) |
| T+P | n = 10 | - 9 mice euthanized due to tumor size humane endpoint (D+60, 63, 69, 81, 84, 90)  - 1 mouse euthanized due to non-tumor related health concerns (D+78) |
| eFc-HALA-TLR7 | n = 10 | - 7 mice euthanized due to tumor size humane endpoint (D+54, 69, 72, 78, 81, 87, 90)  - 1 mouse found dead (D+138) |
| Per-TLR7 | n = 10 | - 5 mice euthanized due to tumor size humane endpoint (D+54, 78, 99)  - 1 mouse euthanized due to ulcerating tumor lesion (D+69)  - 3 mice euthanized due to bodily fluid accumulation (D+12, 15, 21) |

Figure 5B.

**Supplemental Table 2**

*In vivo* ADC + ISAC combination efficacy study design

| **Treatment group** | **Number of mice per group** | **Event Time and Details** |
| --- | --- | --- |
| T-DXd | n = 10 | - 3 mice euthanized due to tumor size humane endpoint (D+96, 108, 150)  - 6 mice either found dead or euthanized due to bodily fluid accumulation (D+81, 105, 114) |
| T-DXd + ISAC | n = 10 | - 1 mouse euthanized due to tumor size humane endpoint (D+87)  - 1 mouse euthanized due to ulcerating tumor lesion (D+60) |
| T-DXd + Pert | n = 9* | - 1 mouse euthanized due to tumor size humane endpoint (D+105)  - 1 mouse found dead (D+87)  - 1 mouse euthanized due to bodily fluid accumulation (D+144) |
| T-DXd + Per-TLR7 | n = 10 | - 2 mice euthanized due to tumor size humane endpoint (D+99, 111)  - 5 mice euthanized due to bodily fluid accumulation (D+66, 105, 129, 138) |
| Triple Comb | n = 10 | - 1 mouse euthanized due to tumor size humane endpoint (D+108)  - 1 mouse found dead (D+12) |

Figure 6A.

All mice were housed in C. bovis free vivarium at the University of Michigan throughout the study. Mice were randomly divided into groups based on the experimental design, then injected with cells to form tumors, and treated when the size reached 250 mm^3^. Untreated mice served as control group. The tumor volumes were measured using digital calipers and width^2^ × length/2

equation, so no blinding was used. All mouse tumor measurements were included until the mice

reached a humane endpoint. Tumor volumes and weights were monitored for 150 days or until

the humane endpoints set by the Unit of Laboratory Animal Medicine (e.g., tumor volume

exceeding 2000 mm^3^, ulceration greater than 50 % of the tumor surface or active bleeding, and

weight loss greater than 20 % of initial treatment).

*9 mice were used in T-DXd + Pert group due to one mouse found dead before treatment was administered.

**Supplemental Table 3**

AUC analysis on efficacy studies. For statistics, one-way ANOVA was used after AUC analysis.

**Supplemental Figures**

**Figure S1.** Selection of a moderate-potency TLR7 agonist for in vivo studies. A first-generation TLR7 agonist was used in NJH-395 that was approximately 3-fold more potent than R848 (Janku et al. 2022, Cancer Immunol Res.). However, NJH-395 suffered from on-target toxicity including cytokine release syndrome (CRS) and anti-drug antibody (ADA) responses. Lower potency has the potential to reduce some effects like ADA responses (Chen et al. 2023, Journal of Immunological Methods). This is seen clinically with lower potency payloads like that used in BDC-1001 enable high dosing (e.g. 20 mg/kg every 2 weeks) with little CRS or ADA reported. However, the potency may not be sufficient for efficacy with or without combination treatment. A moderate potency TLR7/8 agonist was selected for the current studies with intermediate potency relative to NJH-395 and BDC-1001 payloads and commercial availability for benchmarking novel compounds.

**Figure S2.** ADC and ISAC combination efficacy study and weight loss in syngeneic mouse model. 4T1 tumor bearing Balb/c mice were treated with EphA2-PBD plus EphA2 carrier antibody, EphA2-TLR7 (DAR2), or EphA2-TLR7 (DAR 8). While ADC combined with ISAC (DAR 2) showed enhanced responses than unconjugated carrier antibody, high DAR ISAC combination led to severe weight loss in the mice, causing early termination of the treatment.

**Figure S3. T-DXd Bystander Effects with Pertuzumab Combination.** Tumors resected from NCI-N87 bearing nude mice treated with 6.4 mg/kg T-DXd with 19.2 mg/kg pertuzumab were sectioned and stained with γH2A.x antibody to assess the bystander effect of DXd payload. Despite the limited tumor penetration of the ADC (green), DNA damage (red) was observed in cells that were not bound with ADC, indicating a bystander effect. Active blood vessels are shown in blue. Scale bar represents 500 μm.
